# Fetal Cardiovascular Magnetic Resonance: History, Current Status, and Future Directions

**DOI:** 10.1002/jmri.29664

**Published:** 2024-11-23

**Authors:** Dominika Suchá, Anneloes E. Bohte, Pim van Ooij, Tim Leiner, Eric M. Schrauben, Heynric B. Grotenhuis

**Affiliations:** ^1^ Department of Radiology and Nuclear Medicine University Medical Center Utrecht Utrecht The Netherlands; ^2^ Department of Pediatric Cardiology Wilhelmina Children's Hospital Utrecht The Netherlands; ^3^ Department of Radiology and Nuclear Medicine Amsterdam University Medical Center Amsterdam The Netherlands; ^4^ Department of Radiology Mayo Clinic Rochester Minnesota USA

**Keywords:** fetal imaging, cardiovascular, magnetic resonance imaging, congenital heart disease

## Abstract

Fetal cardiovascular magnetic resonance imaging (MRI) has emerged as a complementary modality for prenatal imaging in suspected congenital heart disease. Ongoing technical improvements extend the potential clinical value of fetal cardiovascular MRI. Ascertaining equivocal prenatal diagnostics obtained with ultrasonography allows for appropriate parental counseling and planning of postnatal surgery. This work summarizes current acquisition techniques and clinical applications of fetal cardiovascular MRI in the prenatal diagnosis and follow‐up of fetuses with congenital heart disease.

**Level of Evidence:**

3

**Technical Efficacy:**

Stage 3

## History

The prevalence of congenital heart disease (CHD) at birth has been estimated at 1787 cases per 100,000 newborns worldwide in 2017.^1^ As treatment options have improved, early and precise prenatal CHD diagnosis has become increasingly important, allowing for more advanced surgical corrections and even fetal surgical interventions. Correct prenatal diagnostics also allow for appropriate parental counseling with a significant positive impact on parental stress during and after pregnancy. Delivery planning and anticipated postnatal care are key to improving survival and reducing mortality. The increase in CHD prevalence at birth (+4.2% over the last two decades), the differences in prevalence between low‐income and high‐income countries (2.8% vs. 5.6%), and the significant decrease in related mortality (−45% to −20%) underline the importance of prenatal screening policies and accurate detection tools.^1^, ^2^ CHD screening is typically performed at 18–24 weeks gestation by echocardiographic imaging, although earlier assessment is becoming more common.

Since its introduction for fetal imaging in the mid‐1960s, echocardiography has been the key assessment tool and the first‐choice imaging modality in current guidelines. Echocardiography is safe, readily available, and can be performed early in pregnancy. However, its performance is dependent on hardware, readers' experience and expertise, and is further challenged by several conditions including maternal obesity, presence of uterine fibroids, abdominal wall edema or scar, oligohydramnios, unfavorable fetal position, twins, and heterotaxy. The overall reported sensitivity and specificity of echocardiography for CHD are 69%–74% and 99%–100%, respectively.^3^, ^4^ The accuracy of prenatal diagnosis also depends on the gestational age (GA) and pretest probability, with sensitivities ranging from 45% in low‐risk to 89% in high‐risk populations and about 45%–66% in unselected risk cohorts.^3^, ^5^ Detection rate differs by the type of CHD pathology, as echocardiography often provides acceptable four‐chamber views for cardiac screening, but assessment of the outflow tract and measurement of vessels and blood flow can be problematic.^6^ For example, hypoplastic left heart syndrome (HLHS) with a clear absence or hypoplasia of left heart structures is typically depicted in a four‐chamber view, which explains the high detection rates in most centers. In contrast, outflow tract anomalies are still missed in 39%–68% of screening cases^6^, ^7^, ^8^ and total anomalous pulmonary venous return remains undetected in 88%–98% of cases.^9^, ^10^

Over the past decades, magnetic resonance imaging (MRI) has emerged as a complementary modality for prenatal imaging, in addition to fetal echocardiography. Already in 1983, first attempts were made to obtain fetal MRI during the third trimester of pregnancy.^11^, ^12^ These preliminary studies focused on obtaining the fetal head diameter and crown‐rump length on a 0.04–0.08 T magnet. From 1984 until the late 1990s, studies evaluated which fetal anatomical structures could be depicted using 0.15–0.35 T MRI.^13^, ^14^, ^15^ Image quality was hampered by low spatial resolution and noise, as well as by gross fetal motion, for which commonly transplacental sedation was administered.^14^ At that point, MRI could identify the fetal heart from 15 to 25 weeks' gestation, often as a low‐signal intensity image void. The intracardiac structures and large vessels could be identified during the third trimester in some cases, but images remained blurry and largely nondiagnostic.^16^ Delineation of cardiac structures remained unreliable even in the late 1990s, mainly due to fetal cardiac motion, while ex vivo MRI of the fetal heart by then offered a more detailed anatomical assessment and added value to postmortem examination.^17^

Fetal cardiovascular MRI began to show clinical potential when MRI techniques in general started to improve in the 2000s. By 2005, for the first time, real‐time cine fetal cardiovascular MRI allowed functional cardiac fetal imaging and the estimation of ventricular volumes in the fetus.^18^, ^19^, ^20^ The relatively low temporal resolution, together with high fetal heart rates and related motion artifacts, however, continued to limit functional cardiovascular assessment. For postnatal cardiovascular imaging, techniques emerged that improved temporal and spatial resolution by adjusting for cardiac and respiratory motion. Adapting these techniques for prenatal imaging proved to be challenging. It is not possible to control gross fetal motion without sedation, and obtaining a reliable cardiac gating signal (referred to as an electrocardiography [ECG] signal) from the small rapidly beating heart in a MRI environment has only recently become possible. Techniques to synchronize fetal heart rates with data acquisition started to emerge from 2010 onward and include both indirect‐ECG gating and, more recently, direct ECG‐gating techniques (further discussed below). Nevertheless, accurate assessment of cardiac function and vessel dimensions remains challenging. Additional techniques to correct for maternal and gross fetal motion have been evaluated over the last decade.^21^ In addition to cine imaging, the introduction of motion correction offered the possibility to employ two‐dimensional phase contrast (2D‐PC) imaging in a prenatal setting to assess fetal blood flow^22^, ^23^ and, in combination with T_2_‐mapping, the measurement of fetal oxygen saturation (MR oximetry).^24^, ^25^, ^26^ This is important, as fetal circulation has been shown to impact the development of other organs such as the lungs and the brain, with the latter mostly relying on cerebral perfusion and oxygen supply. Hence, MRI is a promising technique for concomitant fetal heart and brain assessment and a potentially valuable prognostic tool. In light of these strengths, fetal cardiovascular MRI was added to the 2014 American Heart Association (AHA) fetal cardiac disease guidelines as a complementary imaging modality for the “evaluation of visceroatrial situs, venous return, and associated extracardiac malformations (class IIa/C), with *future* potential for the assessment of cardiac structure and ventricular volume and function (IIb/B).”^27^

In the 8 years following the publication of the AHA guideline statement, ongoing improvements in spatiotemporal resolution now allow for better delineation of small fetal structures and extend the potential clinical value of MRI. Nevertheless, fetal cardiovascular MRI has mainly been performed and evaluated for research purposes over the past two decades. Only recently a few specialty centers worldwide have started dedicated fetal cardiovascular MRI imaging as an adjunct clinical modality for fetal assessment. Even though the latest technical advances have shown to be promising, several challenging obstacles are still to be overcome in clinical practice. In this review paper, we summarize the current acquisition techniques, clinical applications, and hurdles to widespread clinical implementation of fetal cardiovascular MRI in the prenatal diagnosis and follow‐up of fetuses with CHD.

## Current Status

### Part One: Fetal Cardiovascular MRI Techniques

**FIGURE 1:**
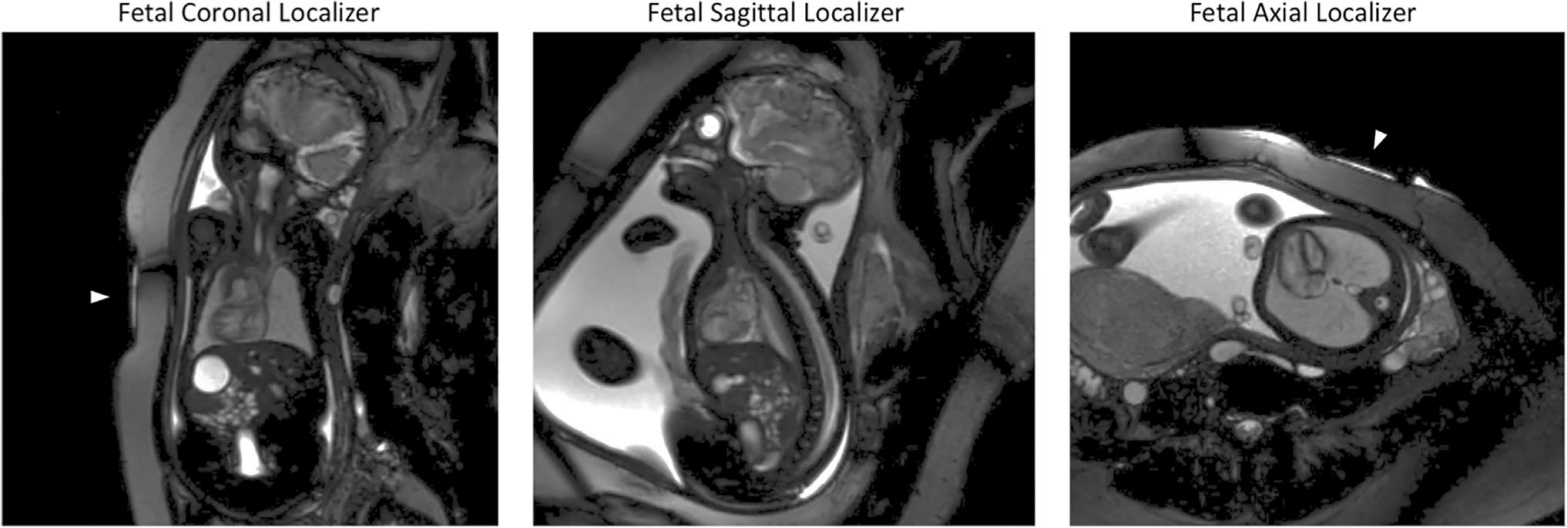
Presented are three planning localizers in fetal coronal, sagittal and axial orientation obtained at 1.5 T MRI in a fetus at gestational age of 36 weeks. Arrowhead shows the position of the Doppler ultrasound probe and the hyperintense signal from the ultrasound gel. Figure courtesy of Erin Englund and Alex J. Barker (Department of Radiology, Pediatric Radiology University of Colorado, Anschutz Medical Campus, USA)

As alluded to above, many challenges exist with fetal cardiovascular MRI. High spatial and temporal resolution MRI is required to characterize the small size of fetal cardiac structures (2–30 mm),^28^ at the high fetal cardiac frequency of 120–160 beats per minute.^29^ Motion of the fetus also plays an outsized role as bulk fetal motion occurs relatively randomly and is convolved with maternal respiratory motion. Finally, maternal anatomy requires a relatively large field‐of‐view acquisition to avoid wrap‐around artifacts. An example of fetal cardiovascular MRI localizers is presented in Fig. 1.

Fetal imaging has evolved rapidly, and most studies have been performed at 1.5 T MRI. The use of higher field MRI (3 T), however, has become more widespread over the last few years and provides better signal‐to‐noise ratios and higher spatial resolution.^30^ This is particularly advantageous when combining fetal heart and brain imaging, which, increasingly, is performed at 3 T MRI. A recent European survey showed a preference for 3 T in 65%–89% of neurological assessments and in 50% of cardiac and vascular indications.^31^ Higher field strength imaging may provide higher image quality in earlier GA, i.e. second trimester imaging,^32^ although this is not supported by the European Society of Pediatric Radiology task force recommendations.^31^ Disadvantages of 3 T imaging include the related increase in field inhomogeneities and banding and shading artifacts, which without proper correction will result in significantly reduced image quality.^30^, ^33^ In Table 1 an overview is provided of common fetal cardiovascular MRI sequences and their utility to detect CHD. As detailed below, most fetal MRI sequences are modified implementations of standard “off‐the‐shelf” techniques. These modifications typically aim to image faster (through, eg, higher parallel imaging factors) to freeze motion and image at higher spatial resolution. Safety concerns related to field strength are discussed below in the dedicated Safety section. In response to the increased field inhomogeneities and artifacts at higher field imaging, and following adult cardiac MRI trends and developments, mid‐field (0.55 T) fetal cardiovascular MRI is being explored currently as well.^34^, ^35^

**TABLE 1:** Fetal Cardiovascular MRI Utilities, Acquisition Types, and Enabling Technologies

Fetal Cardiac Utility	Enabled by	Pros	Cons
Diagnostic anatomical images	Fast (~500 msec per image) nongated acquisition; bSSFP “bright” blood for cardiac structures, T_2_w “black” blood for vascular anatomy	Easy to plan and instant available on MRI scanner; Fast enough to freeze gross fetal movement	Limited in diagnostic utility: no dynamic information, 2D
Diagnosis of vascular abnormalities	Fast multislice acquisitions coupled with slice‐to‐volume reconstructions (SVR)	Volumetric assessment	No dynamic information; rigid SVR not optimized for cardiac applications; long postprocessing
Cardiac dynamic visualization/measurement	Image‐based metric optimized gating (MOG)	Scanning irrespective to heart rate; freely available online	May fail with significant variations in RR interval duration; requires postprocessing
	Doppler US gating	Plug‐and‐play; can be used with any cardiac gated sequence	Significant upfront cost; expertise for transducer placement
	Cardiac self‐gating with radial acquisition	Similar to MOG—continuous acquisition	Extensive postprocessing and corrections for radial sampling

#### NONGATED ACQUISITION

**FIGURE 2:**
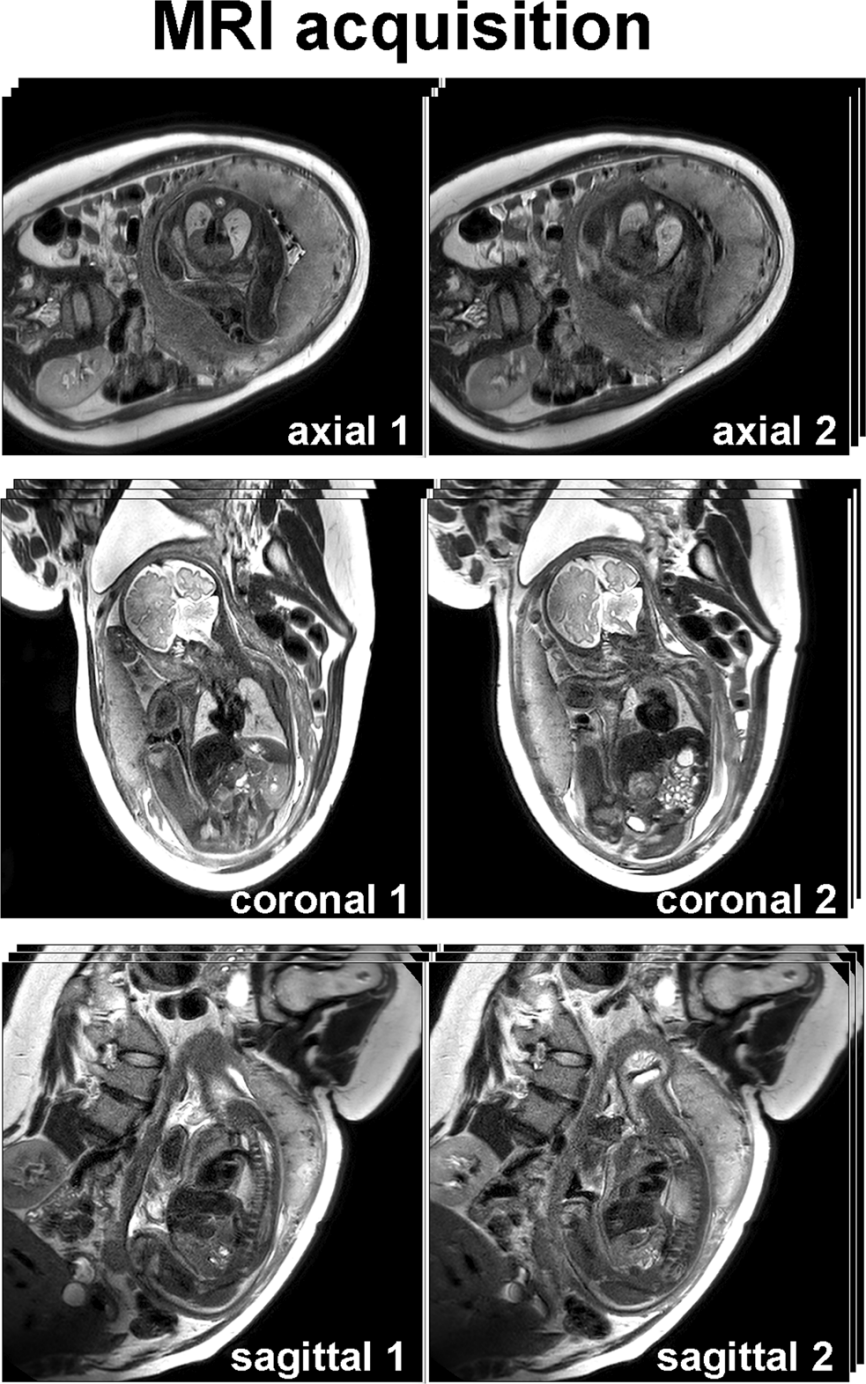
Presented are two axial, two coronal, and two sagittal stacks obtained in a T_2_‐weighted black blood acquisition at 3.0 T MRI. These acquisitions delineate the fetal position and its gross anatomy, and they can be used to reconstruct three‐dimensional volumes as presented in Figure 3.

Rapid acquisition of nongated images can be used to reduce motion artifacts and provide a diagnostic assessment of the fetal vasculature and gross anatomy. Typically, nongated acquisition protocols comprise “bright blood” imaging by balanced steady‐state free precession sequences (bSSFP) and “black blood” imaging by T_2_‐weighted single‐shot fast spin‐echo sequences.^36^ Bright blood acquisition sequences are inherently fast because of the short repetition time (TR) of bSSFP and can employ parallel imaging acquisitions, while black blood images utilize the combination of half‐Fourier k‐space coverage and turbo spin‐echo. The advantage of these black blood sequences is the relatively short acquisition time of approximately 500 msec together with a high spatial resolution of 1–2 mm.^20^, ^37^ Multislice bright blood images can provide information on cardiac structures, while black blood images are helpful for the evaluation of vascular anatomy, as with the latter vessels will appear as low‐signal intensity flow voids due to signal loss from flowing blood. For the assessment of anatomical structures, typically the three canonical planes are obtained comprising axial, coronal, and sagittal views (Fig. 2). Four‐chamber axial views, in general, allow for anatomical assessment without the need for more time‐consuming cardiac‐specific views.^38^ Multiplanar assessment particularly improves visualization of the outflow tracts and large vessels.^39^ Nongated (either cine or multislice) bSSFP sequences may be used to assess cardiac axes and overall cardiac anatomy and ventriculoarterial connections, especially from the mid‐second trimester onward.^33^, ^39^ Using a noncontrast angiography modified time‐of‐flight sequence has been proposed for bright blood vessel visualization.^40^ Movie S1 in the Supplemental Material shows a free‐breathing, real‐time, four‐chamber cine acquisition.

#### NONGATED 2D TO 3D RECONSTRUCTIONS

**FIGURE 3:**
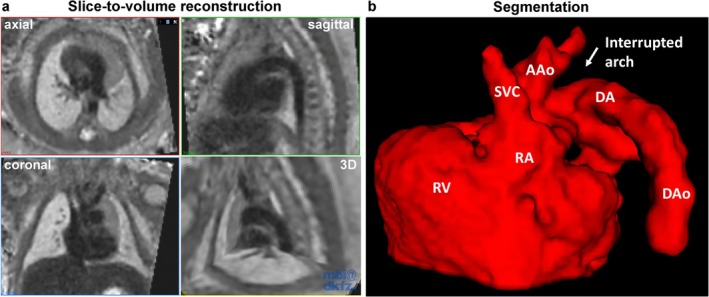
(**a**) Six stacks obtained in three directions (as presented in Fig. 2) are used to reconstruct a volume showing the anatomy of the fetal heart and its thoracic vasculature. (**b**) From this, a 3D segmentation model can be created that shows the anomaly: an interrupted aortic arch. AAo = ascending aorta; DA = ductal arch; DAo = descending aorta; RA = right atrium; RV = right ventricle; SVC = superior vena cava.

While fast acquisition sequences are helpful to reduce gross bulk motion artifacts within the slice (in plane), nongated or static acquisitions are still prone to motion artifacts and reduced image quality. Moreover, nongated acquisitions do not warrant structural continuity between slices (through plane), which is required for volumetric assessment and spatial orientation. Motion correction by slice‐to‐volume registration (SVR) postprocessing techniques has been evaluated, providing three‐dimensional (3D) volume reconstructions from a stack of motion distorted 2D‐images.^41^ Recently, these (rigid) SVR techniques have mainly been used for motion correction of the geometrically static fetal brain. For structures subject to geometrical deformation such as the heart and chest, deformable SVR (DSVR) methods have been proposed more recently^42^ and used to develop 3D anatomical chest and cardiovascular atlases.^43^, ^44^ These techniques are promising but not yet commercially available, and clinical validation is needed.^45^ Figure 3 shows an example of SVR reconstruction of fetal cardiac T_2_‐weighted images.

#### FETAL CARDIAC GATING

To overcome cardiac motion artifacts and allow for dynamic imaging, data acquisition must be synchronized to the cardiac cycle. In contrast to the strong ECG signal of the mother, the ECG signal of the fetus is relatively weak, and obtaining it reliably in an electromagnetic field environment is not possible. Current techniques for cardiac‐gated imaging comprise metric optimized gating (MOG), Doppler ultrasound (DUS) gating and cardiac self‐gating.^46^, ^47^, ^48^

**FIGURE 4:**
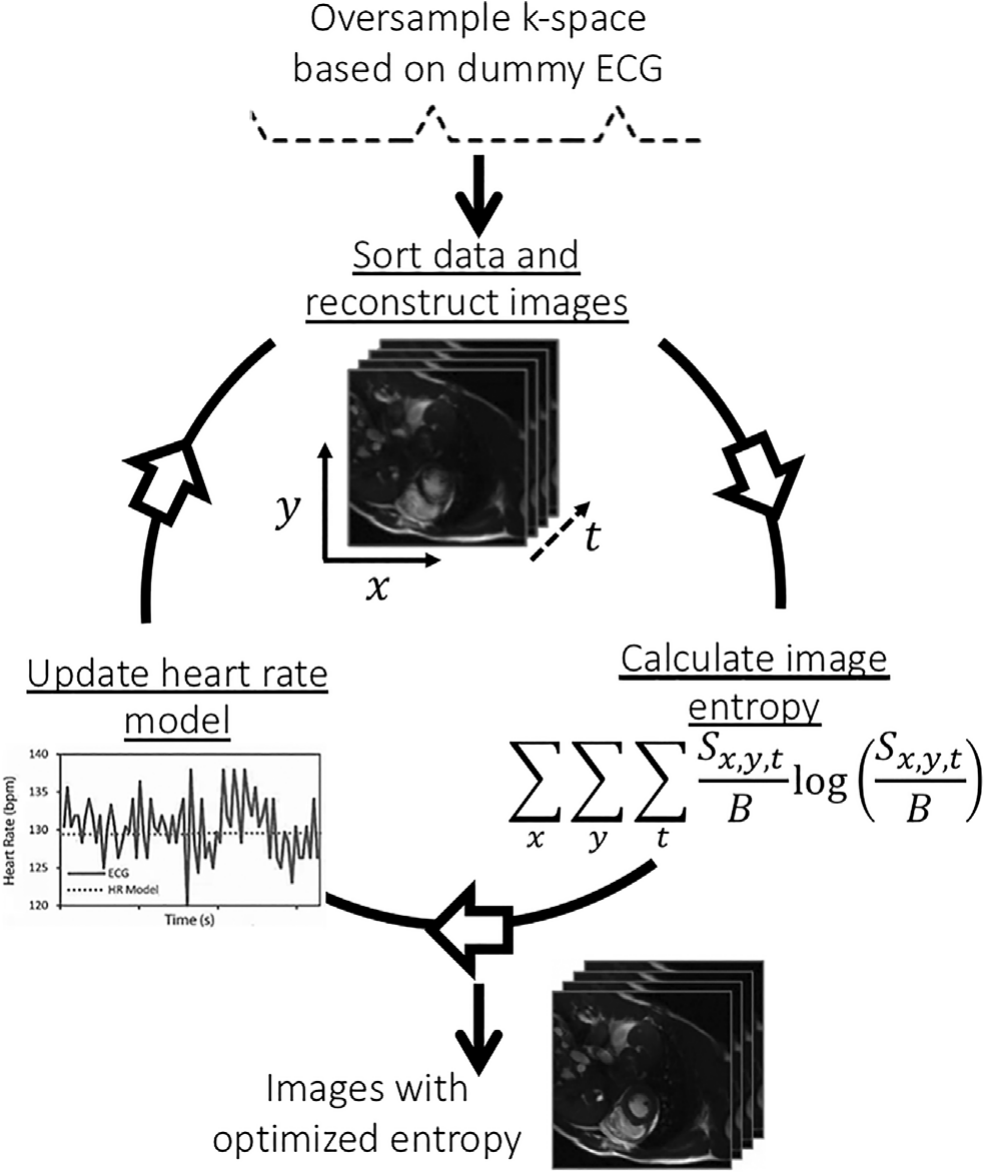
Schematic overview of metric optimized gating (MOG) based on image entropy calculations.

MOG was originally developed to improve blood flow assessment by 2D‐PC imaging, but it can also be applied for static and dynamic (cine) SSFP acquisition.^49^ The technique is based on oversampling the cardiac cycle, thus obtaining excessive data for each k‐space segment.^46^, ^49^ Oversampling ensures that all cardiac phases and the full range of expected fetal heart rates are covered. By using a parameterized model of the fetal heart rate in postprocessing, data are retrospectively sorted according to every theoretical cardiac phase. Acquired data are reconstructed into an image, and by detecting typical misgating artifacts using an image entropy metric, the cardiac phase error can be defined and corrected for. This process is repeated until entropy is minimized (Fig. 4). Although regular heart rates are easy to predict and model, MOG may be more challenging in cases of significant heart rate variations during acquisition, possibly leading to less optimized image results. Image reconstruction was previously time‐consuming, requiring up to several hours, but has improved more recently with increased computing power. Nonetheless, MOG has proven to be useful in a number of studies investigating cardiac function and the quantification of fetal blood flow.^22^, ^50^, ^51^

Cardiac‐gated imaging by DUS relies on real‐time fetal heart rate assessment by an MRI‐compatible ultrasound transducer placed on the mother's abdomen, measuring the Doppler signal of the fetal heart wall.^48^, ^52^ This device directly measures the fetal heart rate in real‐time, and its signal is used as an external gating device input for the MRI system. Thus, data acquisition is synchronized simultaneously without the need for laborious postprocessing. This also allows for the direct adjustment of unexpected heart rate variations during data acquisition and evaluation of slice position and image quality. A limiting factor may be the cost of the device, and currently, only one manufacturer offers Food and Drug Administration (FDA) and European Medicines Agency (EMA) approved MRI‐compatible transducers for this purpose (Smart‐sync®, Northh Medical, Hamburg, Germany). One of the technical challenges of this gating technique is the need for correct placement of the transducer on the abdominal wall in relation to the fetal position (see also Fig. 1). Furthermore, gross fetal motion or maternal breathing may disturb this relation and might require repositioning of the transducer,^52^ significantly prolonging, if not doubling, the overall examination time.^53^, ^54^, ^55^ The need for repeated acquisitions due to trigger signal loss was described in 24% of healthy and almost 50% of suspect CHD clinical fetal cardiovascular MRI cases recently.^56^, ^57^

Other cardiac synchronization methods comprise cardiac self‐gating techniques, which rely on the extraction of gating signals directly from the acquired image data itself.^58^ By extracting MRI signals that characterize cardiac contraction, including echo‐peak magnitude, kymogram or 2D‐image correlation methods, data can be retrospectively sorted by cardiac phase.^59^ These techniques can, however, be unreliable mainly because of the small size and rapid motion of the fetal heart.

#### ACCELERATION TECHNIQUES

Many accelerated fetal imaging methods rely on continuous golden angle radial sampling MRI.^60^ With radial sampling, the k‐space is filled in a spoke wheel matter, contrary to the more conventional parallel phase encoding (Cartesian) sampling method. The advantage of radial sampling is that trajectories are less prone to motion artifacts since data are sampled in varying frequency‐encoding directions, and measurements are averaged as every spoke passes the k‐space center. The use of golden angle radial sampling ensures the k‐space is covered evenly at any time point. Hence, data acquisition is continuous and can be used for dynamic assessment. The main disadvantages of radial sampling are the relative undersampling of the k‐space periphery, the greater reconstruction time, and the presence of eddy currents and streaking artifacts. Eddy current artifacts may be reduced by tiny golden angle sampling.^47^ To overcome significant undersampling and streaking artifacts, radial acquisitions need to be combined with more effective acceleration techniques.^61^

Acceleration techniques are important methods for motion artifact reduction in general, and particularly helpful for dynamic cardiac assessment. Common acceleration techniques are based on k‐space undersampling (with both radial and Cartesian sampling methods) and are often combined with compressed sensing and parallel imaging, such as with k–t SPARSE‐SENSE and iGRASP,^61^, ^62^ or with MOG.^63^ Another method to deal with motion artifacts is the correction for in‐plane artifacts and rejection of data with through‐plane artifacts,^64^ or outliers.^65^ A combination of the abovementioned acceleration and reconstruction techniques has shown to potentially allow for self‐gated real‐time cine MRI and the acquisition of four‐dimensional (4D) cardiac MRI in clinical setting,^65^, ^66^, ^67^ and possibly for whole‐heart 3D cine imaging.^68^ An example of novel potential acceleration techniques yet to be validated include super‐resolution image enhancement methods that are based on a combination of phase encoding undersampling with super‐resolution convolutional neural networks.^69^

#### 2D PHASE CONTRAST

The combination of one or more acceleration techniques is vital to accurately assess the fetal heart. Accelerated imaging, cardiac gating, and motion correction are also important for reproducible fetal phase‐contrast (PC) acquisition and flow quantification. PC‐MRI is the gold standard for blood flow and velocity assessment after birth.^36^ A 2D‐PC in the fetus may be performed by Cartesian or golden‐angle radial sampling, in combination with MOG or DUS.^70^, ^71^, ^72^, ^73^ A 2D‐PC remains challenging due to the required small voxel size (eg, 1.3 × 1.3 × 5.0 mm) and gross fetal movement. Motion‐corrected radial PC‐MRI showed higher image quality compared to repeated Cartesian acquisitions and may therefore be the preferred method for fetal PC‐MRI.^50^ Flow results are typically indexed to fetal weight, which can be estimated from SSFP fetal body volume segmentation.^22^, ^74^ Based on fetal flow volume distribution estimates and derived flow continuity equations, flow volumes may be calculated when direct flow measurements are not feasible.^75^, ^76^, ^77^

#### 4D FLOW EMERGING/EXPERIMENTAL METHODS

**FIGURE 5:**
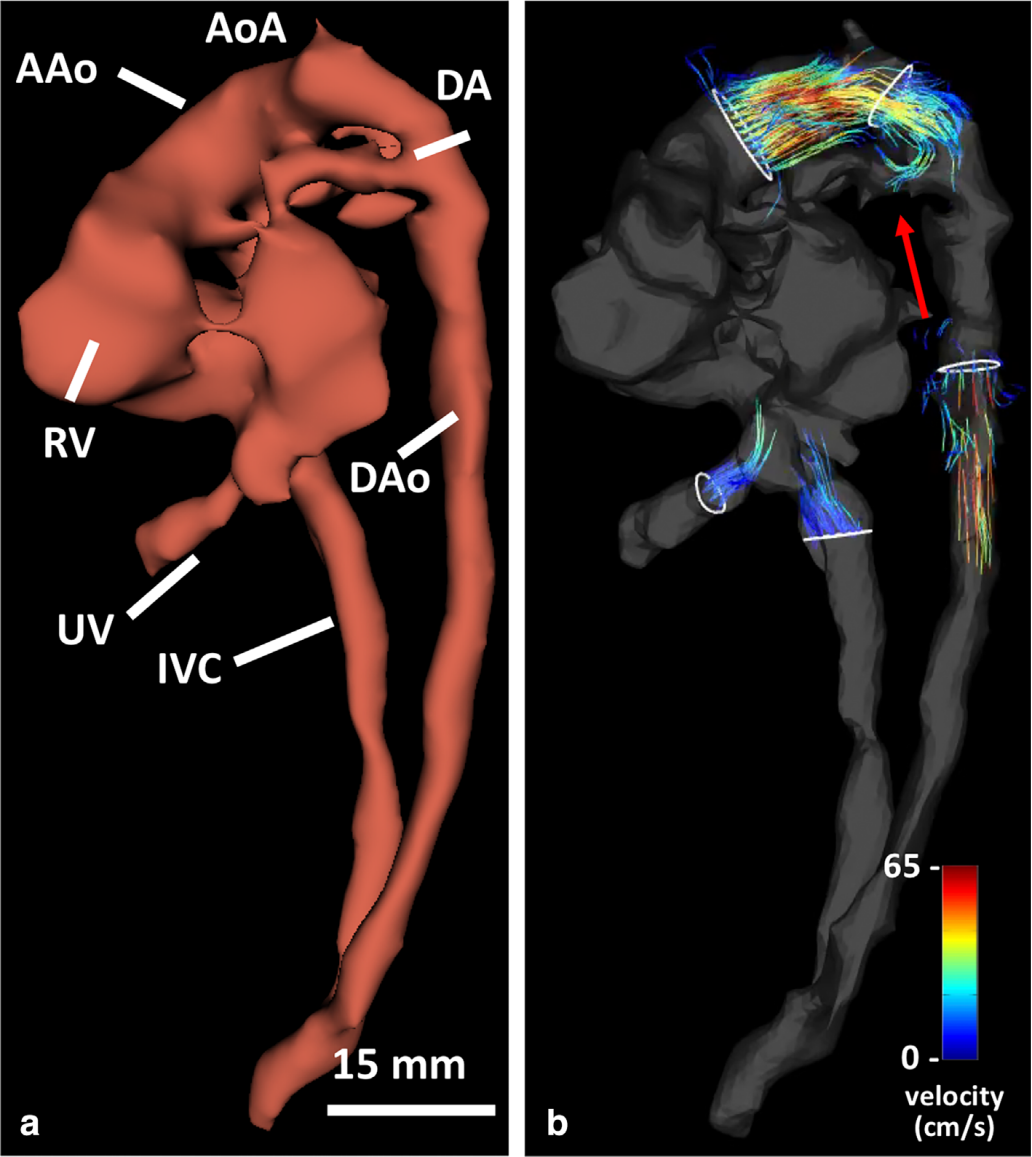
Four‐dimensional (4D) flow fetal cardiovascular MRI in a fetus with transposition of the great arteries. (**a**) 3D segmentation of fetal vasculature from the 4D‐flow derived phase contrast angiogram. (**b**) Time‐resolved pathlines (shown at 128 msec from the cardiac trigger) emitted from the denoted vessels, with visible diastolic reverse ductal flow (red arrow). Note the caliber of the aortic arch and descending aorta, helpful to exclude interruption or coarctation. An animated version of these pathlines can be found in Movie S2 in the Supplemental Material. AAo = ascending aorta; AoA = aortic arch; DA = ductus arteriosus; DAo = descending aorta; IVC = inferior vena cava; MPA = main pulmonary artery; RV = right ventricle; UV = umbilical vein.

Recently, a case series explored the feasibility of 4D flow MRI together with direct cardiac gating by DUS and compressed sensing at 3 T for the assessment of large artery flow patterns and flow quantification.^55^ A 4D‐MRI based velocity map is shown in Fig. 5 and Movie S2 in the Supplemental Material. Results on whole heart 4D blood flow cine MRI are promising but require validation and larger studies.^78^ In addition to flow quantification, flow measurements can be used to calculate the oxygen transport in the fetal large vessels.^25^, ^77^ With MR oximetry, fetal hematocrit and oxygen saturation can be assessed using T_1_‐weighted and T_2_‐weighted imaging and the calculation of the paramagnetic effect of deoxyhemoglobin by T_2_‐relaxation times.^79^, ^80^ As recently shown in vivo, the assessment of fetal oxygen saturation is feasible, but due to T_2_‐measurement variability, it may not yet be precise enough for clinical use to differentiate pathology.^80^, ^81^ By combining oximetry and flow measurements, the distribution of blood flow and oxygen in the fetus may be assessed. An example of such assessment is presented in the recent study by Lee et al.^82^ For oximetry, maternal breath hold during acquisition may be used to limit gross fetal motion.

#### SAFETY

The main safety concerns with fetal MRI have been the temperature increase by radiofrequency energy and specific absorption rate (SAR) and potential related effects on fetal morbidity and mortality, as well as MRI‐related acoustic injury. To prevent harm, the International Commission on Non‐Ionizing Radiation Protection limits SAR to 4 W/kg maternal weight averaged over 30 minutes,^83^ which is now default in all current MRI systems, irrespective of field strength. Until now, no detrimental effects to the fetus have been found after MRI exposure in all trimesters of pregnancy.^84^, ^85^, ^86^ With respect to gadolinium‐based contrast media, contrast‐enhanced imaging should be omitted unless it is critical for maternal or fetal health.^87^ So far, contrast‐enhanced imaging does not play any role in fetal MRI. Long acquisition times may cause maternal discomfort and may increase the chance of fetal movement. There is ongoing debate about the supine vs. left lateral decubitus maternal position and the effect on venous return and placental perfusion.^88^, ^89^, ^90^ For MR examination durations of up to 45 minutes, no maternal discomfort differences have been found, and factors other than position seem to contribute to potential hypotension syndrome symptom development.^88^ Oxygen consumption showed to remain preserved in healthy fetuses.^90^ The International Society of Ultrasound in Obstetrics and Gynecology recommends a patient‐comfortable position and to consider sedation to reduce fetal movements, especially with anxious or claustrophobic patients.^91^ Patient size (>125 kg) may be a relative contra‐indication.^41^

### Part Two: Fetal Cardiovascular MRI in Clinical Setting

Fetal cardiovascular MRI relies on newly developed techniques and adjusted acquisition and reconstruction techniques that are based on well‐established postnatal CMR techniques, as well as on the knowledge and experience gained from imaging animal models. Until recently, published studies mostly focused on exploring the feasibility of proposed fetal cardiovascular MRI techniques and reporting initial experiences regarding the assessment of cardiovascular anatomy or function in the fetus. Most studies have been conducted using 1.5 T magnets in fetuses at a mean GA of over 30 weeks, leveraging the reduced gross fetal motion and larger anatomical dimensions typically present in the third trimester. The reported results are acquisition protocol specific and often derived by institutions and readers who have advanced experience in fetal cardiovascular MRI. Study limitations typically include small sample size (often less than 40 cases), retrospective study design, selected populations, heterogeneous CHD pathology, biased reading, and a lack of reproducibility assessment or no correlation to echocardiography. Hence, study results have to be interpreted with caution and may not be reproducible in different settings or other populations. An overview of fetal cardiovascular MRI in clinical setting is discussed below and presented in Table 2.

**TABLE 2:** Fetal Cardiovascular MRI in Clinical Setting

Indication	Target	Acquisition	IQ^a^	Anatomy	Additional Value^b^	Pathology^b^
Structural	Cardiac	Static bSSFP four‐chamber view	++	Cardiac and apex position	Detection of normal heart	Prediction CHD
		Dynamic nongated bSSFP	++	Myocardial and chamber delineation	Cardiac axis measurement	Malposition
		Gated cine bSSFP^c^	+++		Measurement cardiac dimensions	Cardiomegaly
		T_2_w multiplanar imaging	+/++			Structural defects
						Intracardiac tumors; localization and size
						Tissue characterization
	Great vessels	T_2_w multiplanar imaging	++	Outflow tract	Localization and development of large vessels	Detection of outflow tract anomalies, eg, aortic coarctation, interrupted arch
		T_2_w additional views; eg, transverse aortic arch	+++	Aortic isthmus/arch	Measurement of aortic isthmus and SVC	Show perinatal changes of aortic arch anomalies, eg, double arch transition
		SVR (3D) motion‐corrected reconstructions	+++	Descending aorta		Detection of venous vascular anomalies, eg, SVC and venous return anomalies
		Dynamic nongated bSSFP	++	Pulmonary arteries		
		Gated cine bSSFP^c^	+++	Superior vena cava (SVC)		
				Pulmonary venous return		
	Extra‐cardiac	T_2_w Multiplanar imaging	++	Trachea	Normal organ development, position, and measurements	Tracheal narrowing/compression, eg, in aortic arch anomalies
		SVR (3D) motion‐corrected reconstructions	+++	Lungs	Mediastinal shift (angle)	Lung anomalies, eg, lymphangiectasia, hypoplasia
		Dynamic (non)‐gated bSSFP	++	Mediastinum		Diaphragmatic hernia
				Diaphragm		
		Combined with fetal brain acquisition series^d^	NA	Brain and ventricles	Normal development, gyrification	CHD‐related impaired neurodevelopment
					(A)symmetries	Structural lesions, eg, tuberous sclerosis complex disease
					Dimensions and volumes of structures including ventricles	
Functional	Cardiac	Dynamic nongated multislice bSSFP	++	Ventricular contours/volumes	Ejection fraction estimation	Abnormal dimensions
		Gated cine bSSFP^c^	+++	Myocardial delineation	Cardiac output calculation	Impaired cardiac function
					Feature tracking and strain analysis	Potential prognostic marker
						Potential to differentiate between CHD and controls or between prognoses based on strain differences
	Circulation	2D‐phase contrast (PC)	++	Cardiac chambers	Circulation distribution patterns	Detection of outflow tract obstruction
		Gated‐PC^c^	+++	Great vessels	Flow measurement	Circulation/flow alterations in CHD, including pulmonary and cerebral flow
		4D flow	+++	Ductus arteriosus	Oxygen saturation measurement	Shunt calculations, including foramen ovale
		T_1_w and T_2_w imaging/mapping	++	Umbilical vessels	Combined ventricular output (CVO) calculation	Saturation and lack of oxygen delivery in CHD
					Plan atrial septal interventions	Predictive value, eg, in perinatal aortic arch anomaly development, neurodevelopmental outcomes
						Guide maternal hyperoxygenation therapy
						Evaluate (pharmacological) treatment effect on shunts

bSSFP = balanced steady‐state free precession; CHD = congenital heart disease; IQ = image quality; NA = not assessed; SVR = slice‐to‐volume registration.

^a^ Image quality rated based on overall literature results from worst (+) to best (++). Note: IQ results are institutional acquisition protocol specific and rely strongly on reader experience.

^b^ To echocardiography, especially when echocardiography is limited or inconclusive.

^c^ Doppler ultrasound (DUS), metric optimized gating (MOG), or “self”‐gated acquisition.

^d^ Beyond the scope of this review.

#### STRUCTURAL CARDIOVASCULAR ASSESSMENT AND DIAGNOSIS OF CHD

**FIGURE 6:**
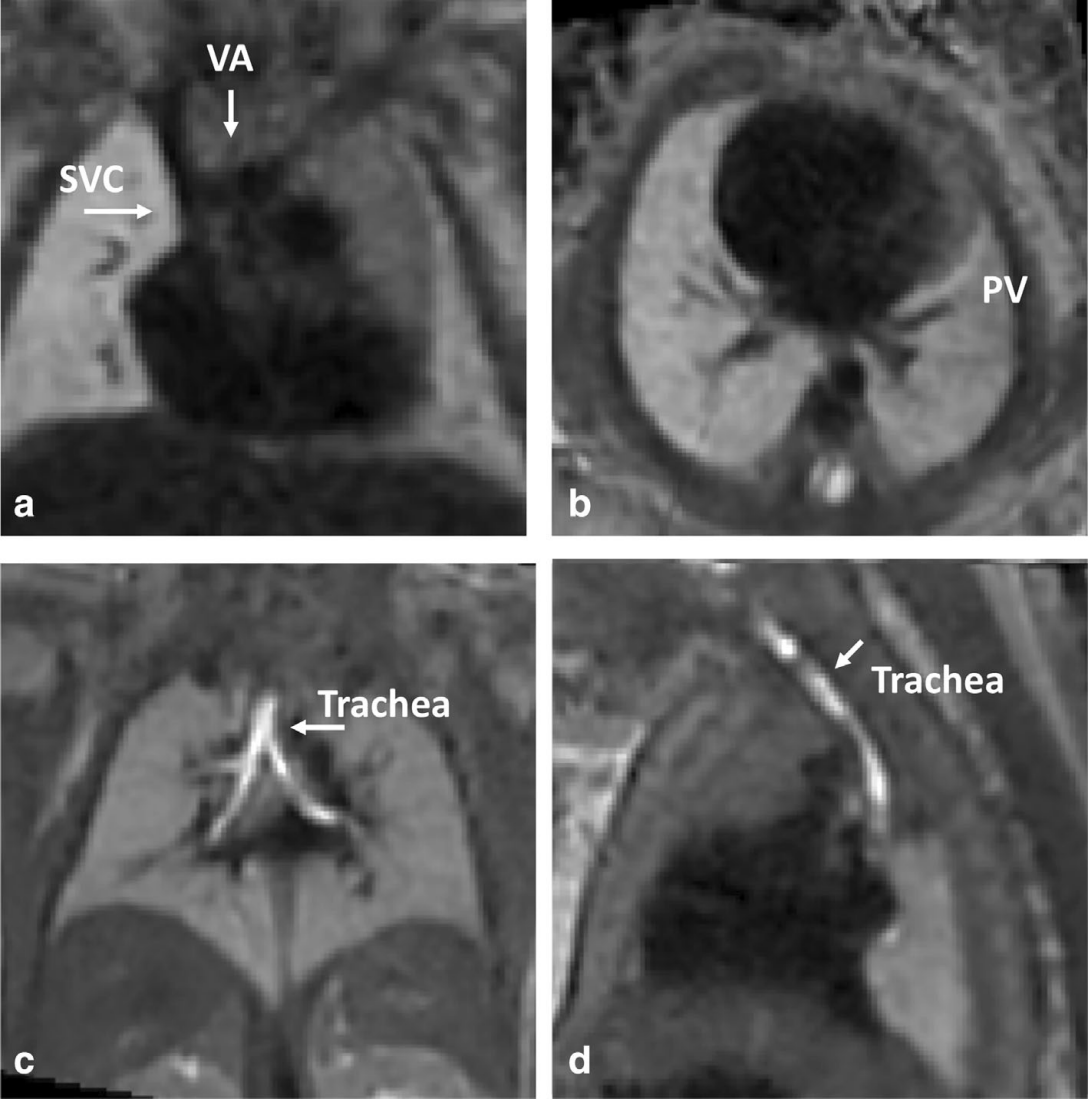
Fetal cardiovascular MRI in a fetus GA 33+ weeks allows assessment of (**a**) normal superior vena cava (SVC) and vena anonyma (VA) connections, and (**b**) normal pulmonary vein (PV) anatomy. (**c**, **d**) A fetus of GA 34+ weeks shows the development and caliber of the airways evaluated in the same 3.0 T MRI T_2_‐weighted acquisition, as well as the overall aspect of the lungs and normal position of liver (c).

**FIGURE 7:**
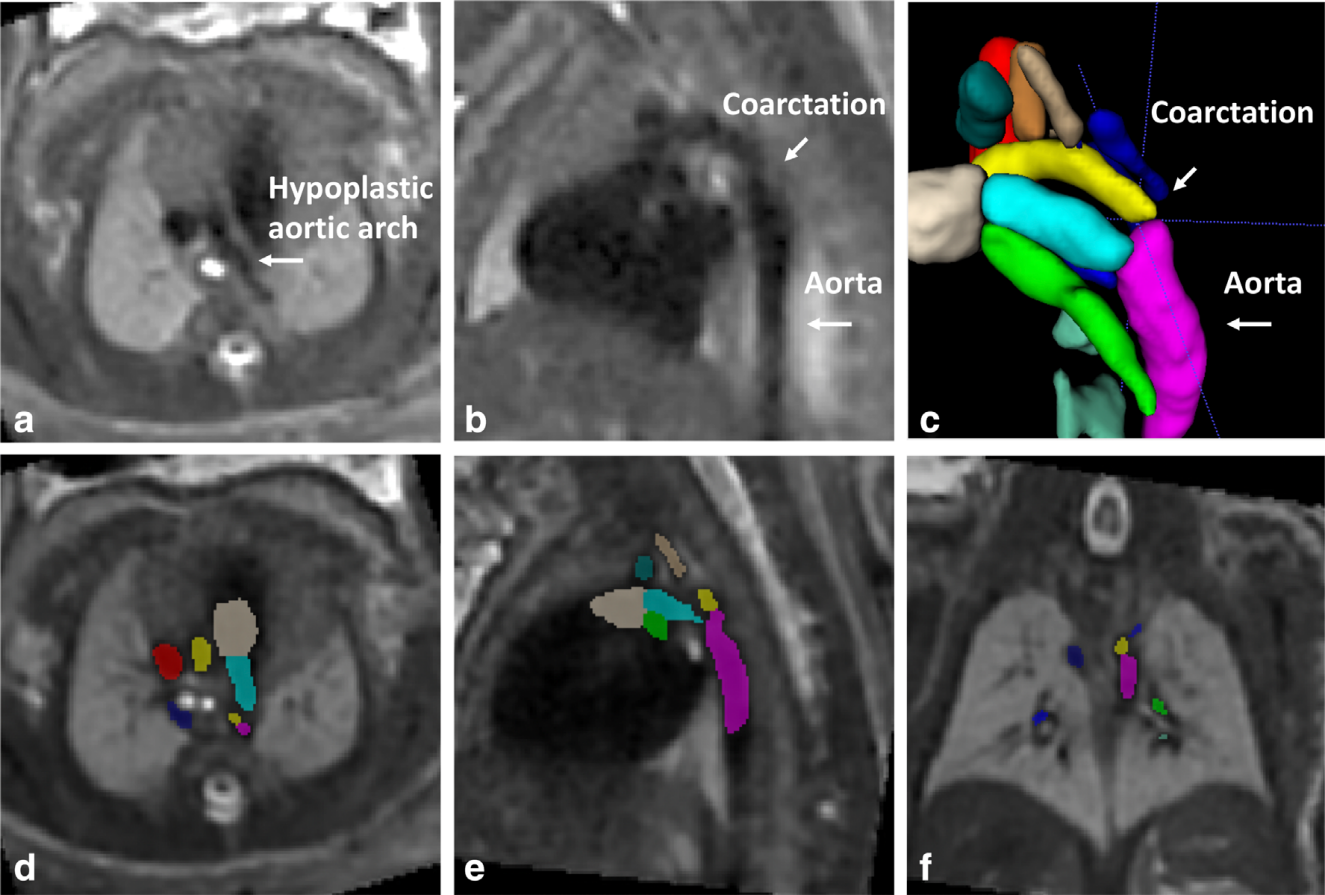
Fetal cardiovascular MRI in a fetus GA 34+ weeks with hypoplastic left heart syndrome obtained at 3.0 T MRI. Presented are: (**a**) a hypoplastic aortic arch and (**b**) aortic coarctation with poststenotic dilatation of the descending aorta. (**c**) Shows the aortic coarctation in the 3D reconstruction, which is based on vessel segmentation presented in (**d**–**f**). Movie S4a,b in the Supplemental Material show the excellent image quality for vascular (venous) assessment.

Static bSSFP fetal cardiovascular MRI has shown to be able to delineate basic cardiac and great vessel anatomy^19^, ^20^, ^38^, ^39^, ^92^, ^93^, ^94^, ^95^ as typically assessed in CHD following the (modified) sequential segmental analysis approach.^39^, ^96^ In general, better visualization was found for anatomical structures presented in four‐chamber views compared to structures requiring outflow tract, aortic arch, or venous return assessment.^93^ Four‐chamber views yielded reliable measurements of the cardiac axis and apex position, demonstrating good interobserver reproducibility and agreement with echocardiography,^39^, ^97^ with an 85% sensitivity and 81% specificity for predicting the presence of CHD.^97^ Multiplanar imaging and using additional views such as the transverse aortic arch enhanced the detection of outflow tract and vascular anomalies.^98^, ^99^, ^100^, ^101^, ^102^, ^103^, ^104^ Three‐dimensional motion‐corrected fetal cardiovascular MRI reconstructions significantly improved the visualization of vascular structures compared with 2D images (97% vs. 53% of structures visualized, respectively) and showed excellent interobserver correlation for descending aorta, transverse arch, and superior vena cava vessel measurements,^41^, ^105^ as well as good agreement with echocardiography (mean measurement bias −0.33 mm).^41^ An example of vascular assessment is presented in Fig. 6a,b. The 3D motion‐corrected reconstructions also allowed for discrimination of aortic coarctation from false positive cases in a cohort of 108 fetuses with normal ventriculoarterial connections using statistical vascular shape modeling (0.907 area under the receiver operating characteristic),^106^, ^107^ although further validation is needed. Furthermore, motion‐corrected 3D images were able to show the evolution of double aortic arch pathology and related perinatal changes of arch anomalies.^108^ A clinical coarctation case with hypoplastic arch and additional vessel and 3D segmentations is presented in Fig. 7.

**FIGURE 8:**
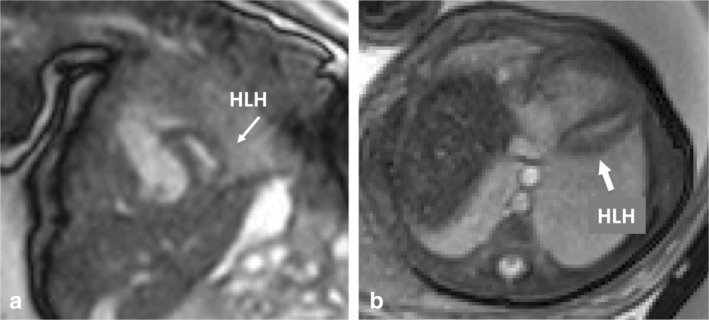
Two examples of fetal 3.0 T balanced steady‐state free precession (b‐SSFP) cine images acquired with Doppler ultrasound (DUS) gating. (**a**) Fetus GA 35 + 3 with hypoplastic left heart (HLH) showing paradoxical septal motion on short axis views (Movie S3a in the Supplemental Material). (**b**) HLH example in axial view (Movie S3b in the Supplemental Material).

Dynamic (nongated) fetal cardiovascular MRI, when compared with static techniques, enabled improved anatomical visualization,^33^, ^109^, ^110^ especially for anomalies related to the outflow tract and aortic arch.^33^, ^111^ However, even with dynamic imaging, reliable assessment of pulmonary arteries, pulmonary veins, and atrioventricular valves remains relatively challenging.^33^, ^67^, ^109^ Dynamic bSSFP demonstrated similar interobserver agreement and comparable results to echocardiography in cases with complete anatomic coverage and minimal motion artifacts.^109^ DUS‐gated dynamic (cine) imaging may further enhance the visualization of cardiovascular structures and improve myocardial delineation (Fig. 8; Movie S3a–c in the Supplemental Material).^52^, ^54^, ^56^ For aortic isthmus diameter measurements, DUS‐gated cine bSSFP showed good agreement with echocardiography (10.8% variability, −2.3% bias).^53^

**FIGURE 9:**
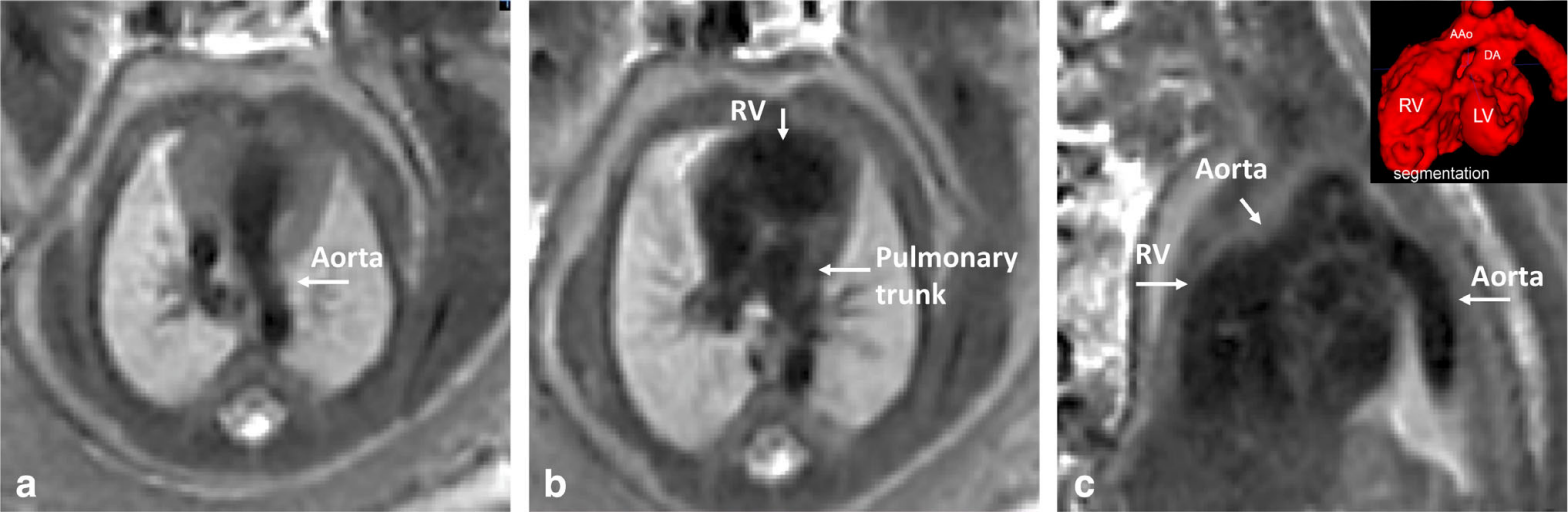
Fetal cardiovascular MRI in a fetus GA 34+ weeks with dextro‐transposition of the great arteries (d‐TGA). Presented are the ventriculoarterial (VA) discordant connections; (**a**, **c**) aorta in anterior right position from the right ventricle, (**b**) caudal from the aorta arises the pulmonary trunk in posterior left position from the left ventricle (LV, shown in 3D). (**c**, upper right) Shows the d‐TGA in a 3D segmentation model. Movie S5 in the Supplemental Material shows all axial images in which both ventricles are shown in normal position and a ventricular septal defect is present. Note the excellent image quality for vascular (venous) assessment.

Diagnostic fetal cardiovascular MRI has been reported in various types of cardiac and large vessel abnormalities.^104^ Clinical studies evaluating fetal cardiovascular MRI accuracy for CHD diagnosis, however, are scarce. The performance of fetal cardiovascular MRI for CHD diagnosis has mainly been evaluated retrospectively in patients with proven or suspected CHD at fetal echocardiography with postnatal confirmation. fetal cardiovascular MRI readers were often not blinded to echocardiography results, which hampers evaluation of true diagnostic test accuracy. Reported results vary depending on the type of cardiovascular CHD, with numbers of correct diagnosis by fetal cardiovascular MRI ranging 58%–100%.^93^, ^100^ Most recently, Vollbrecht et al conducted a prospective diagnostic study with DUS‐gated cine bSSFP imaging at a mean 36 weeks gestation age for the detection of abnormal cardiovascular characteristics and showed comparable accuracy to echocardiography (sensitivity, 91.8% vs. 93.6%, specificity, 99.9% vs. 99.9%, respectively) and a reliable diagnosis of complex CHD in 21/23 (91%) cases.^57^ We present a clinical case of HLHS in Fig. 8 and Movie S3 in the Supplemental Material. Other studies have highlighted the additional value of fetal cardiovascular MRI particularly for the assessment of large vessels and detection of vascular anomalies: 1) in aortic arch or ductus arteriosus assessment^95^, ^99^, ^100^, ^103^, ^104^, ^110^, ^112^ with higher anomaly detection rates and improved specificity than echocardiography (96% and 87% vs. 61% and 20%, respectively)^103^, ^112^ and 2) in systemic or venous return assessment^101^, ^102^, ^104^, ^110^, ^112^ with improved detection of persistent left superior vena cava (100% vs. 69% echocardiography).^101^ Examples from our clinic include assessment of normal venous anatomy (Fig. 6; Movies S4 and S5 in the Supplemental Material), detection of aortic arch hypoplasia with aortic coarctation (Fig. 7; Movie S4a,b in the Supplemental Material) and cases of transposition of the great arteries (TGA) (Figs. 5 and 9; Movies S2 and S5 in the Supplemental Material).

Regarding intracardiac pathology, a similar detection rate between fetal cardiovascular MRI and echocardiography has been reported.^113^ More frequently, however, fetal cardiovascular MRI was found to provide complementary information, especially when echocardiography was limited or inconclusive.^54^, ^92^, ^104^, ^114^ The large cohort of Dong et al comprised 1379 fetuses referred for inconclusive echocardiography results, in which fetal cardiovascular MRI performed at a mean 24.5 weeks GA and assessed by readers with 16 years' fetal cardiovascular MRI experience, found correct and incorrect CHD diagnosis in 60% (42/71 fetus) and 39% (28/71), respectively.^104^ Importantly, fetal cardiovascular MRI was able to correctly detect 99% (1265/1275) of normal hearts. Non‐CHD cardiac findings (eg, malposition or cardiomegaly) were accurate in 88% (29/33). In fetuses with ectopia cordis, fetal cardiovascular MRI may complement echocardiography in some cases.^115^ Fetal cardiovascular MRI may also aid in the detection, localization, and size assessment of intracardiac masses such as myxoma or rhabdomyoma and allow for tissue characterization.^104^, ^110^, ^116^

#### FUNCTIONAL CARDIAC ASSESSMENT

**FIGURE 10:**
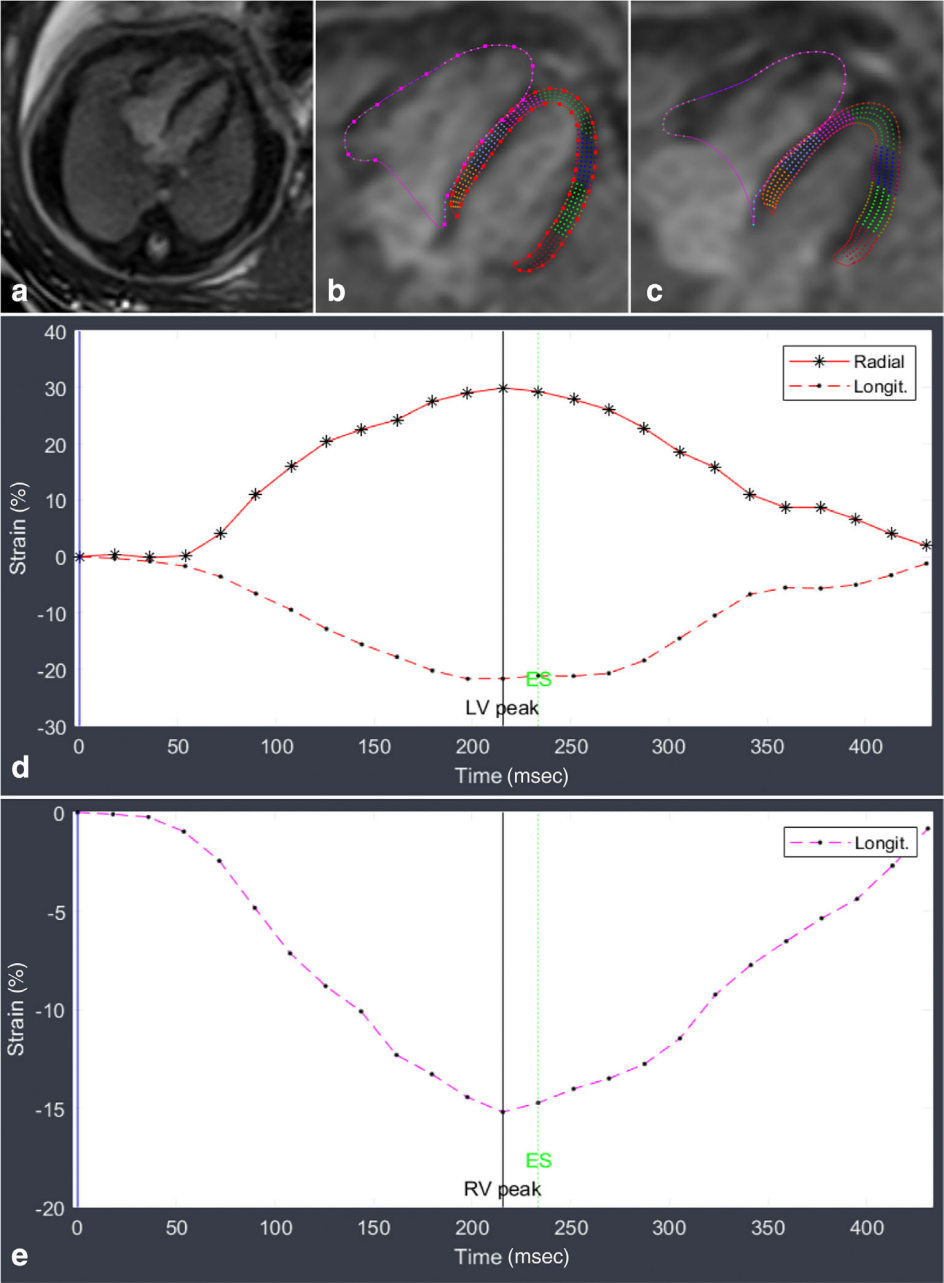
Presented are (**a**) balanced steady‐state free precession (b‐SSFP) cine images acquired with Doppler ultrasound (DUS) gating in four‐chamber view. Myocardial contouring in end‐diastole (**b**) and end‐systole (**c**) allows for assessment of cardiac function and strain analysis of the right ventricle (**d**) and left ventricle (**e**). Figure courtesy of Thomas M. Vollbrecht and Julian A. Luetkens (Department of Diagnostic and Interventional Radiology, University Hospital Bonn, Germany).

Quantification of cardiac function is not yet part of the routine clinical work‐up with fetal cardiovascular MRI. Fogel et al were the first to report functional cardiac measurements assessed in two cases by fetal cardiovascular MRI.^18^ Subsequently, Chaptinel et al measured left ventricular end‐systolic and end‐diastolic areas on short‐axis views in six fetuses as a composite for ejection fraction (EF) and found moderate to good agreement between fetal cardiovascular MRI and echocardiography.^59^ The interobserver bias was larger with fetal cardiovascular MRI compared to echocardiography but showed smaller confidence intervals, indicating greater precision (mean 25.68 mm^2^ [0.01; 51.35] vs. 10.41 [−49.52; 70.34], respectively). Nongated multislice fetal cardiovascular MRI acquisitions showed a significant discrepancy between fetal cardiovascular MRI and echocardiography for EF (mean 48% vs. 76%, respectively, in CHD and 51% vs. 67%, respectively, in controls).^117^ DUS‐gated acquisitions have already shown good interobserver agreement for ventricular volume and myocardial wall thickness measurements with mean interobserver differences of 3.2% end‐diastolic volume (EDV), 3.3% end‐systolic volume (ESV) 5.8% stroke volume (SV), 2.9% (EF) and 4.5% (end‐diastolic (ED) wall thickness).^52^ In a clinical setting, DUS‐gated cine acquisitions or fast nongated bSSF iGRASP sequences have provided good image quality for qualitative ventricular function evaluation in the assessment of biventricular vs. univentricular outcome.^54^ A recent cohort study (*N* = 25) presented initial cine CMR reference values for healthy third‐trimester fetuses.^56^ Ventricular volumes were found to be ≥48% larger than those obtained by echocardiography (using Simpson's method). However, cardiac output fell within the normal echocardiographic *z*‐ranges, and overall interobserver reproducibility was deemed moderate to good. Most recently, DUS‐gated cine acquisitions were used for myocardial delineation and feature tracking for myocardial strain analyses.^118^, ^119^ In these two state‐of‐the‐art studies, strain analyses were successful in 88%–94% of fetal cardiovascular MRI and showed strain differences between CHD cases and controls and between various CHD pathologies. A strain analysis example is presented in Fig. 10.

#### FETAL CIRCULATION AND OXYGEN SUPPLY

**FIGURE 11:**
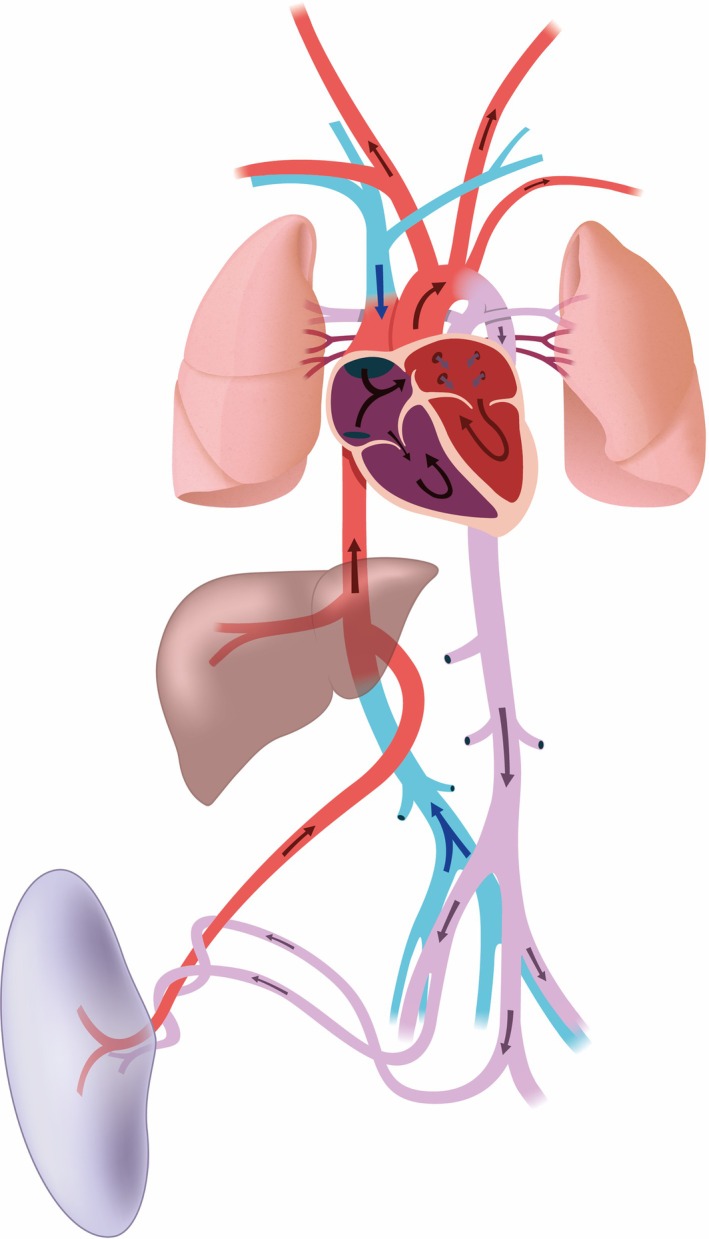
Schematic overview of the fetal circulation and oxygenation. Colors indicate blood oxygenation levels from high (red) to low (blue).

Evaluation of fetal circulation and blood volume distribution is crucial as the development of vascular structures and organs relies on their perfusion and oxygen supply.^75^ Fetal circulation differs from that of the newborn (Fig. 11), and distribution patterns in CHD have been shown to differ from those healthy fetuses. Fetal cardiovascular MRI flow and oxygen saturation measurements in the umbilical vein can provide estimations of fetal oxygen delivery.^82^ However, this does not provide information on the flow and oxygen distribution to individual organs. Flow measurements in the large vessels are more specific and typically include assessment of the main pulmonary artery (MPA), left and right pulmonary artery (LPA and RPA), ascending aorta (AAo), superior vena cava (SVC), ductus arteriosus (DA), aortic arch (AA), and descending aorta (DAo). Pulmonary blood flow can thus be obtained directly from MPA flow measurements or indirectly calculated from LPA and RPA measurements. As an additional measure, the combined ventricular output (CVO) can be calculated,^82^ as well as the foramen ovale (FO) shunt. The 2D‐PC has shown a feasible and reliable tool for flow measurements in large vessels of healthy fetuses,^22^, ^46^ as well as in fetuses with left‐sided CHD.^23^ MOG‐PC has also demonstrated good agreement with conventional 2D‐PC in a phantom study.^51^ Alternatively, DUS‐gated PC fetal cardiovascular MRI has been validated against MOG‐PC and echocardiography for DAo and UV flow measurements.^71^, ^73^ MOG‐PC blood flow reference values have been reported only for late GA fetuses.^72^ PC fetal cardiovascular MRI, along with T_2_‐based oximetry, has been used to obtain flow velocities, flow distribution, and oxygen saturation in comparison to healthy controls in several CHD subtypes, including HLHS and single ventricle physiologies, cyanotic CHD (i.e. TGA), tetralogy of Fallot, and tricuspid valve atresia and anomalies.^25^, ^26^, ^77^, ^80^, ^82^, ^120^, ^121^ CHD subtypes showed significant differences in flow distribution and changes in, for example, pulmonary and cerebral flow. These are important findings that help understand the impact of physiology on fetal growth and development. Reduced flow has been significantly associated witch changes in aortic arch shape, potentially predisposing to postnatal aortic coarctation.^105^ While Fricke et al did not find improved diagnosis of left‐sided cardiac obstructions (HLHS, aortic coarctation), fetal cardiovascular MRI might be used to depict more severe cases of outflow obstruction.^121^ Altered flow patterns in tricuspid valve CHDs have been correlated with neurodevelopmental outcomes.^120^ An earlier study in CHD fetuses already showed a relation between reduced cerebral oxygenation and decreased brain volumes.^25^

#### CONCOMITANT ASSESSMENT OF EXTRACARDIAC FINDINGS AND PATHOLOGY

Fetal MRI may be a valuable tool for the complimentary assessment of extracardiac malformations in fetal cardiac disease.^27^ Concomitant evaluation of the brain in fetuses with suspected or known CHD may reveal structural changes and provide information on impaired neurodevelopment.^122^ Assessment of cardiac and cerebral involvement in suspected tuberous sclerosis complex (TSC) might enable prenatal TSC diagnosis.^116^, ^123^ MRI in CHD fetuses has shown a significantly higher sensitivity for the detection of extracardiac lesions compared with echocardiography (84% vs. 32%, respectively).^113^ In fetuses with abnormal cardiac axes detected by echocardiography, MRI may aid in detecting the cause of cardiac malposition, such as congenital diaphragmatic hernia or lung anomalies^124^ and delineate related (prognostic) findings such as the mediastinal shift (angle).^125^ In HLHS, MRI is used as a screening tool for pulmonary lymphangiectasia since it aids in prognosis prediction and perinatal planning.^54^, ^126^, ^127^ Fetal cardiovascular MRI may detect tracheal compression in aortic arch anomalies.^95^ Figure 6 shows the concomitant assessment of cardiac and extracardiac anatomy. A list of current clinical indications for extracardiac fetal MRI in general has been provided by the American College of Radiology and the Society for Pediatric Radiology.^128^

#### GUIDING FETAL INTERVENTIONS

PC and T_2_‐mapping have demonstrated potential as noninvasive imaging tools for the evaluation of umbilical venous oxygen saturation and pulmonary blood flow to guide maternal hyperoxygenation therapy.^26^, ^82^ PC fetal cardiovascular MRI has also been used to measure and grade the degree of ductus arteriosus shunt and evaluate pharmacological, nonsteroidal anti‐inflammatory treatment effects in fetuses with Ebstein's anomaly.^129^ Furthermore, fetal cardiovascular MRI flow measurements supported the need for atrial septal intervention and were used to measure treatment effects in HLHS and TGA fetuses.^23^, ^130^

#### PERINATAL PLANNING AND OUTCOME PREDICTION

The clinical impact of fetal cardiovascular MRI on patient management, clinical outcome, and cost‐effectiveness remains underinvestigated. Although several studies report fetal cardiovascular MRI being helpful for CHD diagnosis, and clinical decision‐making, systematic, prospective studies are lacking. Ryd et al reported clinical utility of fetal cardiovascular MRI as its use and findings had an impact on parental counseling or patient management in 84% of cases with CHD.^54^ Goncalves et al reported an additional value of fetal cardiovascular MRI in 30% of fetuses with CHD, mainly for diagnosing concomitant extracardiac pathology.^113^

## Future Directions

Future directions in the fetal cardiovascular MRI field include the evaluation of fetal cardiovascular MRI in clinical studies as well as further technical developments. For clinical studies, prospective, well‐designed research is needed to evaluate the role of fetal cardiovascular MRI in diagnosis, prognosis, in therapy, or for delivery planning both in general and for specific CHD subtypes. Structured reading and reporting such as by segmental analysis is recommended. Technical developments involve further advances in acquisition techniques, such as (free‐breathing) real‐time cine imaging, 3D cine imaging and 4D flow imaging. Further improvements in reconstruction techniques include automated 3D DSCVR, advanced motion correction and super‐resolution image enhancement. Novel postprocessing methods may also reduce data interrogation time. The integration of deep learning methods for structure segmentation, classification and modeling is likely to broaden the clinical utility of fetal cardiovascular MRI. Additionally, assessing cost‐effectiveness, postnatal impact on CHD‐care and outcomes, and gaining fetal cardiovascular MRI experience are important aspects to further delineate the clinical utility of fetal cardiovascular MRI and refine its complementary value to fetal echocardiography.

## Supporting information


**Movie S1.** Free‐breathing, real‐time (non‐ECG gated) four‐chamber cine acquisition. Presented is a 3.0 T free‐breathing, real‐time (non‐ECG gated) four‐chamber cine acquisition. Note the maternal anatomy and the required relatively large field‐of‐view acquisition to avoid wrap‐around artifacts.

**Movie S2.** Four‐dimensional (4D)flow fetal cardiovascular MRI in a fetus with transposition of the great arteries. (a) 3D segmentation of fetal vasculature from the 4D‐flow derived phase contrast angiogram. (b) Time‐resolved animated pathlines emitted from the denoted vessels, with visible diastolic reverse ductal flow (red arrow). Note the caliber of the aortic arch and descending aorta, helpful to exclude interruption or coarctation. AAo: ascending aorta; AoA: aortic arch; DA: ductus arteriosus; DAo: descending aorta; IVC: inferior vena cava; MPA: main pulmonary artery; RV: right ventricle; UV: umbilical vein.

**Movie S3.** Doppler ultrasound gated b‐SSSP cines in hypoplastic left heart. Two examples of fetal 3.0 T balanced steady‐state free precession (b‐SSFP) cine images acquired with Doppler ultrasound (DUS) gating. (a) Fetus gestational age (GA) 35 + 3 with hypoplastic left heart (HLH) showing paradoxical septal motion on short axis cine views. (b) HLH shown in axial view cine images.

**Movie S4.** T_2_‐weighted series for vascular and extracardiac anatomic assessment. Fetal cardiovascular MRI in a fetus GA 34+ weeks with hypoplastic left heart syndrome obtained at 3.0 T MRI. Presented are excellent image quality T_2_‐weighted acquisitions in (a) axial view and (b) coronal view. Note the hypoplastic aortic arch and aortic coarctation with poststenotic dilatation of the descending aorta. Also note the excellent image quality for vascular venous assessment. These series also allow for evaluation of the development and caliber of the airways evaluated as well as the overall aspect of the lungs and normal position of the liver.

**Movie S5.** T_2_‐weighted vascular series in dextro‐transposition of the great arteries. Fetal cardiovascular MRI in a fetus GA 34+ weeks with dextro‐transposition of the great arteries (d‐TGA). Presented are T_2_‐weighted 3.0 T axial images showing the d‐TGA anatomy with discordant ventriculoarterial connections: aorta in anterior right position from the right ventricle and caudal from the aorta arises the pulmonary trunk in posterior left position from the left ventricle. Both ventricles are shown in normal position and a ventricular septal defect is present. Note the excellent image quality for vascular (venous) assessment.
