# Supplementary figures and images for: Fetal Cardiovascular Magnetic Resonance: History, Current Status, and Future Directions
Source: J Magn Reson Imaging. 2024 Nov 23;61(6):2357–75. doi: 10.1002/jmri.29664 (PMC12063768; doi:10.1002/jmri.29664)

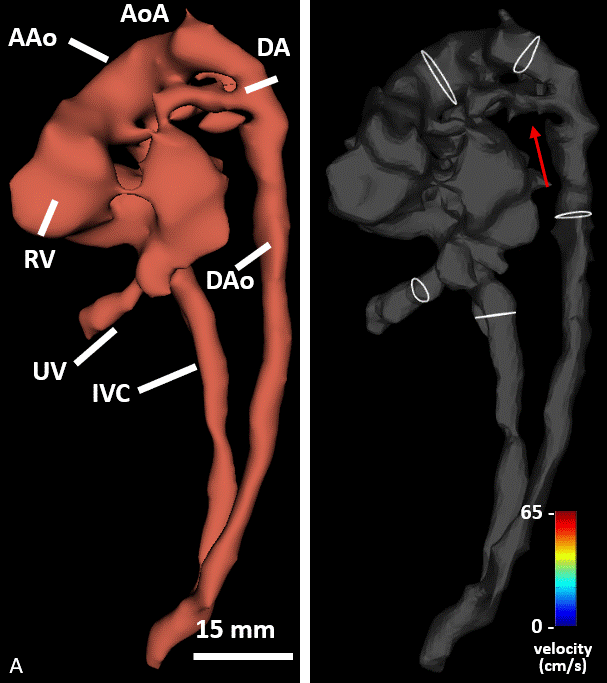

Supplement: Supplementary file 2 — Movie S2. Four‐dimensional (4D)flow fetal cardiovascular MRI in a fetus with transposition of the great arteries. (a) 3D segmentation of fetal vasculature from the 4D‐flow derived phase contrast angiogram. (b) Time‐resolved animated pathlines emitted from the denoted vessels, with visible diastolic reverse ductal flow (red arrow). Note the caliber of the aortic arch and descending aorta, helpful to exclude interruption or coarctation. AAo: ascending aorta; AoA: aortic arch; DA: ductus arteriosus; DAo: descending aorta; IVC: inferior vena cava; MPA: main pulmonary artery; RV: right ventricle; UV: umbilical vein. [file JMRI-61-2357-s001.gif]
